# Autophagy regulates plastid reorganization during spermatogenesis in the liverwort *Marchantia polymorpha*

**DOI:** 10.3389/fpls.2023.1101983

**Published:** 2023-02-09

**Authors:** Takuya Norizuki, Naoki Minamino, Miyuki Sato, Takashi Ueda

**Affiliations:** ^1^ Division of Cellular Dynamics, National Institute for Basic Biology, Okazaki, Japan; ^2^ Laboratory of Molecular Membrane Biology, Institute for Molecular and Cellular Regulation, Gunma University, Maebashi, Japan; ^3^ Department of Basic Biology, SOKENDAI (The Graduate University for Advanced Studies), Okazaki, Japan

**Keywords:** autophagy, plastid, plastid DNA, spermiogenesis, spermatid, spermatozoid, *Marchantia polymorpha*

## Abstract

Autophagy is a highly conserved system that delivers cytoplasmic components to lysosomes/vacuoles. Plastids are also degraded through autophagy for nutrient recycling and quality control; however, the involvement of autophagic degradation of plastids in plant cellular differentiation remains unclear. Here, we investigated whether spermiogenesis, the differentiation of spermatids into spermatozoids, in the liverwort *Marchantia polymorpha* involves autophagic degradation of plastids. Spermatozoids of *M. polymorpha* possess one cylindrical plastid at the posterior end of the cell body. By fluorescently labeling and visualizing plastids, we detected dynamic morphological changes during spermiogenesis. We found that a portion of the plastid was degraded in the vacuole in an autophagy-dependent manner during spermiogenesis, and impaired autophagy resulted in defective morphological transformation and starch accumulation in the plastid. Furthermore, we found that autophagy was dispensable for the reduction in plastid number and plastid DNA elimination. These results demonstrate a critical but selective role of autophagy in plastid reorganization during spermiogenesis in *M. polymorpha*.

## Introduction

Autophagy is a highly conserved mechanism that degrades cytoplasmic components in vacuoles/lysosomes for various cellular functions including nutrient recycling, homeostasis, and reorganization (Morishita and Mizushima, 2019). In plant cells, autophagy participates in various physiological processes such as development and abiotic and biotic stress responses (Marshall and Vierstra, 2018; Norizuki et al., 2020; Su et al., 2020). In addition to bulk degradation for metabolic recycling, a wide range of cytoplasmic material is selectively degraded by autophagy in the vacuole/lysosome. The plastid (chloroplast) is a well-known target of selective autophagy in plant cells, which is important for responses to nutrition-limited conditions, senescence, and chloroplast damage (Izumi and Nakamura, 2018; Zhuang and Jiang, 2019; Wan and Ling, 2022). However, the role of autophagic degradation of plastids in cellular differentiation during plant development remains unknown.

**Figure 1:**
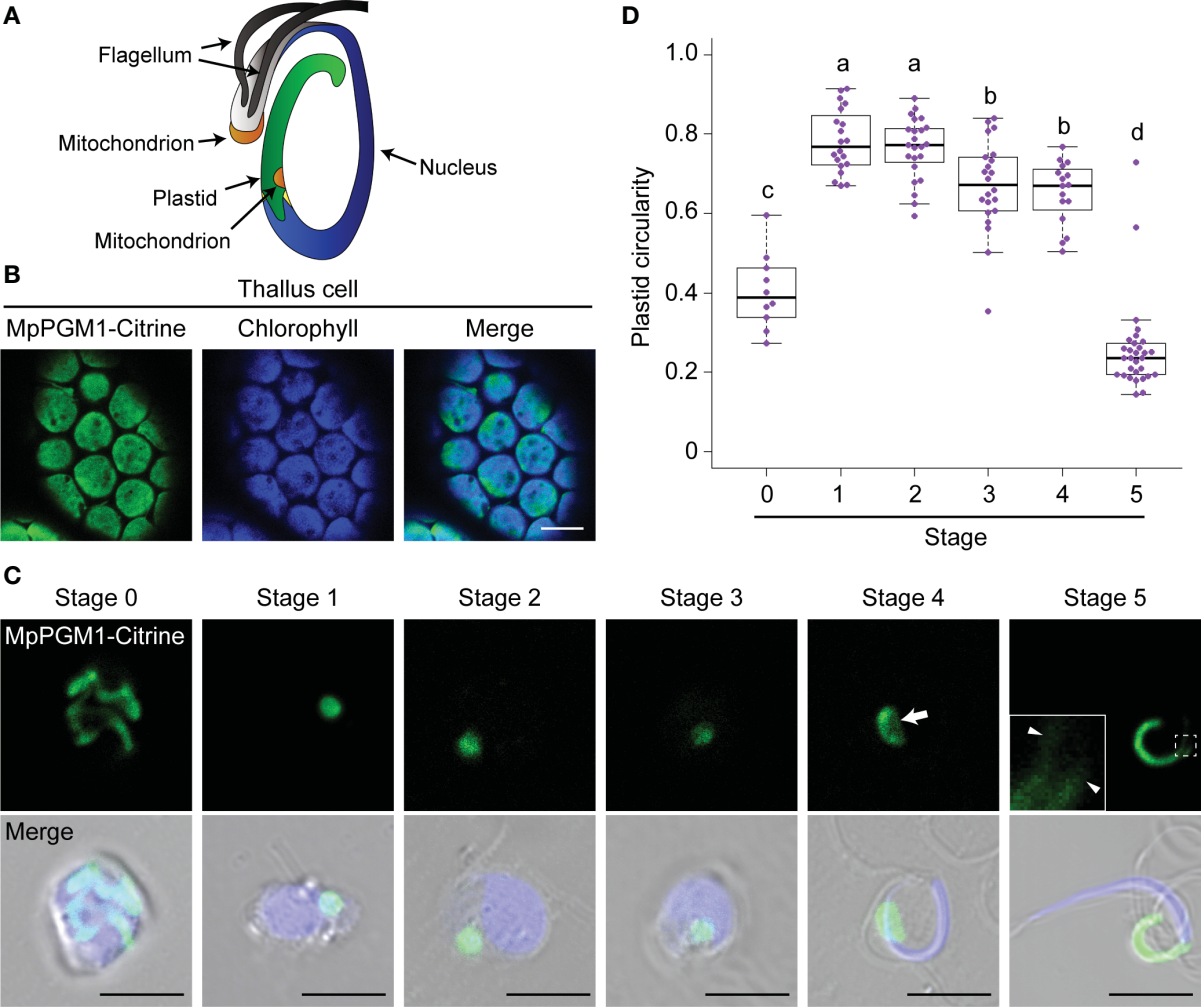
Morphological changes of the plastid during spermiogenesis in *M. polymorpha*. **(A)** Schematic illustration of a spermatozoid of *M. polymorpha*, depicted based on Carothers (1975). **(B)** Confocal images of thallus cells expressing MpPGM1-Citrine (green). The autofluorescence from chlorophyll is shown in blue. Eighteen cells were observed, and a representative result is presented. Scale bar = 10 μm. **(C)** Maximum-intensity projection images of cell-wall-digested antheridial cells and a spermatozoid at each developmental stage expressing MpPGM1-Citrine (green). A total of 20 stage-0, 29 stage-1, 32 stage-2, 31 stage-3, 22 stage-4, and 32 stage-5 cells were observed; representative images are presented. The nuclei were stained with Hoechst 33342 (blue). The arrow and arrowhead indicate the region of the weak signal from MpPGM1-Citrine and tubular extension from the plastid body, respectively. Scale bars = 5 μm. **(D)** The circularity of the plastid was calculated using the same set of samples analyzed in **(C)**. The boxes and solid lines in the boxes indicate the first and third quartiles and the median, respectively. The upper and lower whiskers are drawn at the greatest value smaller than 1.5× the interquartile range (IQR) above the third quartile and the smallest value greater than 1.5× the IQR below the first quartile, respectively. Different letters denote significant differences based on the Steel–Dwass test (*p* < 0.05).

Plastids exhibit a highly pleiomorphic nature during cellular differentiation and environmental changes, especially in non-green plastids (Osteryoung and Pyke, 2014). A striking morphological change in the plastid has also been observed during male gametogenesis in bryophytes. During sexual reproduction, bryophytes produce motile flagellated male gametes, termed spermatozoids. Most bryophyte spermatozoids consist of a cell body containing a cylindrical plastid filled with starch granules, a thin and helically elongated nucleus, two mitochondria, and two flagella protruding from the anterior edge of the cell body (**Figure 1A**; Renzaglia and Garbary, 2001). Transmission electron microscopy (TEM) has shown that plastids change their shape from spherical to cylindrical during spermiogenesis. Drastic reorganization also occurs inside the plastids. In the majority of plant species producing spermatozoids, as spermiogenesis proceeds, starch deposition increases and the thylakoidal structure is diminished, resulting in plastids mostly filled with starch granules (Carothers, 1975; Renzaglia and Garbary, 2001). However, the mechanism by which plastid reorganization occurs remains largely unknown.

Recently, autophagy has been shown to be required for organelle reorganization during spermiogenesis in bryophytes, including the moss *Physcomitrium patens* and the liverwort *Marchantia polymorpha* (Sanchez-Vera et al., 2017; Norizuki et al., 2022). In *P. patens*, impairment of autophagy results in an increased number of plastids without a change in the total area of the plastid (Sanchez-Vera et al., 2017), implying that autophagy suppresses plastid division through an unknown mechanism. However, it remains unclear whether bryophyte spermiogenesis involves autophagic degradation of the plastid and whether morphological transformation of the plastid is somehow mediated through autophagic activity.

In this study, we investigated whether autophagy is required for plastid reorganization during bryophyte spermiogenesis in *M. polymorpha*. Using confocal microscopy, we found that plastids dynamically changed their shape during spermatozoid formation. We also found that a portion of the plastid was degraded in the vacuole *via* autophagy and impairment of autophagy resulted in defective morphological transformation and starch accumulation in the plastid during spermiogenesis. Furthermore, we discovered that plastid DNA was removed independently of autophagy. These results demonstrate that autophagy plays a critical and selective role in plastid reorganization during spermiogenesis in *M. polymorpha*.

## Materials and methods

### Plant materials and growth conditions

*M. polymorpha* accession Takaragaike-1 (Tak-1; Ishizaki et al., 2008) was grown asexually on 1/2× Gamborg’s B5 medium containing 1.4% (w/v) agar at 22 °C under continuous white light. To induce antheridiophores, approximately two-week-old thalli were further cultivated on vermiculite soaked in 1:1000 Hyponex (HYPONeX JAPAN) at 22 °C under continuous white light for two weeks and then cultivated at 22 °C under continuous white light supplemented with far-red light. The transgenic plants used in this study are listed in **Supplementary Table 1**.

### Vector construction and transformation

For nomenclature of genes, proteins, and mutants of *M. polymorpha*, we followed Bowman et al. (2016). Gene IDs were obtained from MarpolBase (http://marchantia.info/), genome version 5.1 (Montgomery et al., 2020). The primer sequences used in this study are listed in **Supplementary Table 2**.

To construct pENTR Mp*PGM1*, CDS for MpPGM1 (Mp4g13750) was amplified using PCR from cDNA prepared from Tak-1 thalli and subcloned into pENTR/D-TOPO (Thermo Fisher Scientific). To construct pENTR Mp*PGM1-mTurquoise2* (*monomeric Turquoise2*), the CDS for mTurquoise2, amplified by PCR, was inserted into the *Asc*I site of the pENTR Mp*PGM1* vector using the In-Fusion HD Cloning System (Clontech). To construct pENTR *TP-sGFP*, the sequence for the transit peptide (TP) of MpSIG2 (Mp4g13380; Kanazawa et al., 2013) was amplified *via* PCR from cDNA prepared from the Tak-1 thalli. The amplified fragments of TP_Mp*SIG2* and cDNA for sGFP were conjugated using PCR *via* homologous double crossover, and the amplified product was subcloned into pENTR/D-TOPO. The sequences flanked by the *att*L1 and *att*L2 sites in pENTR Mp*PGM1*, pENTR Mp*PGM1-mTurquoise2*, or pENTR *TP-sGFP* were introduced into pMpGWB308, pMpGWB103, or pMpGWB303 (Ishizaki et al., 2015), respectively, using the Gateway LR Clonase™ II Enzyme Mix (Thermo Fisher Scientific) according to the manufacturer’s instructions.

Transformation of *M. polymorpha* was performed as previously described (Norizuki et al., 2022). Transformants were selected on plates containing 10 mg/L hygromycin B and 250 mg/L cefotaxime for the pMpGWB103 Mp*PGM1-mTurquoise2* vector, and 0.5 µM chlorsulfuron and 250 mg/L cefotaxime for the pMpGWB308 Mp*PGM1-Citrine* and pMpGWB303 *TP-sGFP* vectors.

### Confocal microscopic and TEM observation

To observe thallus cells expressing MpPGM1-Citrine, five-day-old thalli were used.

To observe stage 0–4 spermatids, antheridia were fixed with 4% (w/v) paraformaldehyde and their cell walls were digested with 1% (w/v) Cellulase Onozuka RS (SERVA) and 0.25% (w/v) Pectolyase Y-23 (Kyowa Chemical Products), as previously described (Norizuki et al., 2022). The samples were incubated in PME buffer (50 mM PIPES, 5 mM EGTA, and 1 mM MgSO_4_, adjusted to pH 6.8 using NaOH) containing 1 µg/mL Hoechst 33342 (Dojindo).

Spermatozoids (stage 5) were obtained by placing a mature antheridiophore upside-down on a drop of 20–30 μL water for 1 min and fixing in 4% (w/v) paraformaldehyde in PBS buffer (150 mM NaCl, 80 mM Na_2_HPO_4_, and 40 mM NaH_2_PO_4_, adjusted to pH 6.8, using NaOH) for 5 min. Fixed spermatozoids were centrifuged at 9,100 × *g* for 1 min, and the pellet was suspended in PBS buffer containing 1 µg/mL Hoechst 33342.

For simultaneous observation of plastid and vacuole markers in spermatids, antheridiophores were manually sectioned using a razor blade.

For immunostaining, fixed and cell-wall-digested antheridial cells were used, as previously described (Norizuki et al., 2022). As a primary antibody, the anti-double-stranded DNA (dsDNA) antibody (HYB331-01; Santa Cruz Biotechnology) was used at a 200× dilution. As a secondary antibody, Alexa Fluor™ 546 goat anti-mouse IgG (H+L) (Thermo Fisher Scientific), or goat anti-mouse IgG (H+L) Alexa Fluor™ Plus 488 (Thermo Fisher Scientific) were used at a 1000× dilution. The samples were mounted using ProLong™ Diamond Antifade Mountant (Thermo Fisher Scientific) and incubated for at least 24 h at room temperature in the dark.

For confocal microscopic observations, an LSM780 confocal microscope (Carl Zeiss) equipped with an oil immersion lens (×63, numerical aperture = 1.4) was used.

For TEM observation, wild-type and Mp*atg5-1^ge^* antheridia were subjected to electron microscopy, as described previously (Norizuki et al., 2022).

### Quantification and statistical analyses

Maximum-intensity projection images created from z-stacked images using ImageJ (version 1.50i; National Institute of Health) were used to calculate plastid circularity. To calculate the occupancy of starch granules in the area of the plastid, the total area of starch granules, which were observed as electron-dense structures, and the area of the plastid were calculated using ImageJ. The number of the large spherical vacuole and the plastid were counted using single-sectioned 22.49 μm × 22.49 μm images. To test the normality of the data, the Shapiro–Wilk test was performed using R (version 3.6.0; The R project), and samples were considered nonparametric when the *p* value was less than 0.05. For comparison between two groups, the Wilcoxon rank-sum test (for nonparametric samples) or Welch’s *t* test (for parametric samples) was performed using R software. For statistical analyses among three or more groups, the Steel–Dwass test was performed using R. Details of the statistical methods are indicated in each figure legend.

## Results

### The plastid changes its shape during spermiogenesis

To observe how the plastid changes its shape during spermiogenesis, we generated transgenic *M. polymorpha* expressing fluorescently tagged MpPGM1. *PGM* genes encode phosphoglucomutase (PGM), and plastidal PGM (AtPGM1) is required for starch synthesis in plastids of *Arabidopsis thaliana* (Caspar et al., 1985; Periappuram et al., 2000). In *M. polymorpha* thallus cells, MpPGM1-Citrine was localized in chloroplasts, overlapping with autofluorescence from chlorophyll, confirming that MpPGM1-Citrine can be used as a plastid marker in *M. polymorpha* (**Figure 1B**). Using this marker, we observed plastid reorganization during spermiogenesis, which can be divided into 1 + 5 stages (stages 0–5) based on flagellar formation and nuclear shape (Minamino et al., 2022). In cells at stage 0, which included spermatogenous cells, spermatid mother cells, and spermatids before the appearance of flagella, tentaculate plastids were observed. At stages 1–4, one plastid with a spherical or lens-like shape was observed in each cell. Notably, at stage 4, an area with a weaker fluorescent signal from MpPGM1-Citrine was detected around the center of the plastid (the arrow in **Figure 1C**), which could be the region where the posterior mitochondrion is embedded, as reported in a previous TEM observation (Carothers, 1975). In stage-5 cells, which are mature spermatozoids, the plastid exhibited a cylindrical shape with thin tubular extensions associated with the posterior end of the nucleus (arrowheads in **Figure 1C**), as described previously (Carothers, 1975). Consistent with this observation, the circularity of the plastid increased between stages 0 and 1 and drastically decreased before stage 5 (**Figure 1D**). Similar results were obtained using another plastid marker, TP-sGFP, sGFP fused with the transit peptide (TP) of MpSIG2, which is a nucleus-encoded subunit of plastid-encoded plastid RNA polymerase (Kanazawa et al., 2013) (**Figure S1**). These results indicate that dynamic morphological changes occur in the plastid during spermiogenesis in *M. polymorpha*.

### Autophagy is involved in the plastid reorganization during spermiogenesis

**Figure 2:**
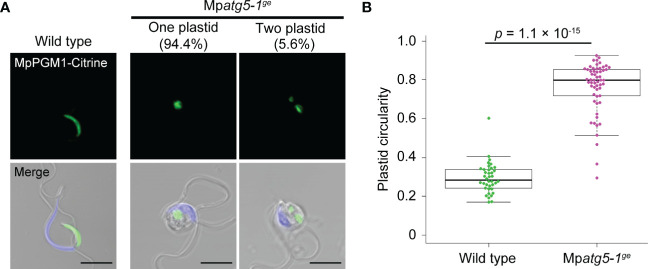
Impaired morphological changes of the plastid in the autophagy-defective mutant. **(A)** Confocal images of wild-type and Mp*atg5-1^ge^* spermatozoids expressing MpPGM1-Citrine (green). The nuclei were stained with Hoechst 33342 (blue). Scale bars = 5 μm. A total of 38 wild-type and 54 Mp*atg5-1^ge^* spermatozoids were observed and representative images are presented. **(B)** The circularity of the plastid was calculated using the same set of samples analyzed in **(A)**. The boxes and solid lines in the boxes indicate the first and third quartiles and the median, respectively. The upper and lower whiskers are drawn at the greatest value smaller than 1.5× the IQR above the third quartile and the smallest value greater than 1.5× the IQR below the first quartile, respectively. The *p* values obtained using the Wilcoxon rank-sum test are presented.

**Figure 3:**
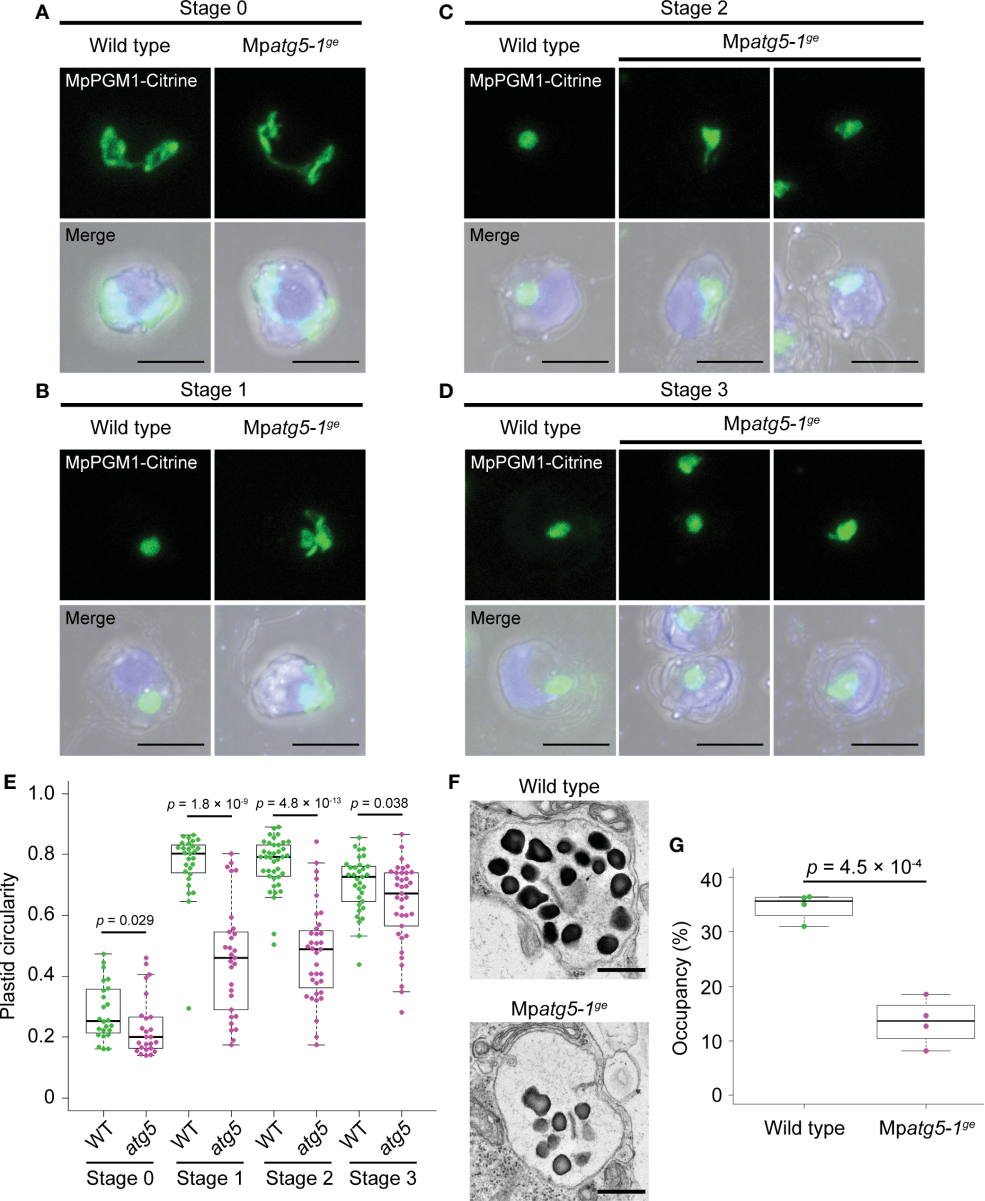
Defects in plastid reorganization during spermiogenesis in the autophagy-defective mutant. **(A–D)** Confocal images of the wild-type and Mp*atg5-1^ge^* antheridial cells expressing MpPGM1-Citrine (green) at stage 0 **(A)**, 1 **(B)**, 2 **(C)**, and 3 **(D)**. Nuclei were stained with Hoechst 33342 (blue). Scale bars = 5 μm. A total of 24 stage-0, 28 stage-1, 39 stage-2, and 33 stage-3 cells were observed for wild-type antheridial cells, and 25 stage-0, 28 stage-1, 35 stage-2, and 37 stage-3 cells were observed for Mp*atg5-1^ge^*. Representative images are presented. **(E)** Circularity of the plastid calculated in the same sets of wild-type (WT) and Mp*atg5-1^ge^* (*atg5*) samples analyzed in **(A–D)**. **(F)** TEM observation of the plastids in wild-type and Mp*atg5-1^ge^* spermatids around stage 1-2. Scale bars = 500 nm. Four plastids were observed for each genotype, and representative images are presented. **(G)** Occupancy of starch granules in plastids. Occupancy indicates the ratio of the total area of the starch granules within a plastid to the area of the plastid. Occupancy was calculated using the same sets of samples analyzed in **(F)**. For **(E)** and **(G)**, the boxes and solid lines in the boxes indicate the first and third quartiles and median, respectively. The upper and lower whiskers are drawn at the greatest value smaller than 1.5× the IQR above the third quartile and the smallest value greater than 1.5× the IQR below the first quartile, respectively. The *p* values obtained by the Wilcoxon rank-sum test **(E)** or Welch’s *t* test **(G)** are shown.

We previously demonstrated that autophagy is required for reorganization of various organelles during spermiogenesis in *M. polymorpha* (Norizuki et al., 2022). To investigate whether autophagy is also involved in plastid reorganization during spermiogenesis, we observed plastids in spermatozoids of the Mp*atg5-1^ge^* mutant, which is defective in autophagy (Norizuki et al., 2019; 2022). Whereas wild-type spermatozoids possessed one cylindrical plastid, more than 90% of Mp*atg5-1^ge^* spermatozoids possessed one spherical plastid, and three out of the 54 Mp*atg5-1^ge^* spermatozoids possessed two spherical plastids (**Figures 2A, B**). This result suggests that morphological changes in plastids involve autophagy. To gain further insight into the role of autophagy in plastid reorganization during spermiogenesis, we observed morphological changes in plastids during spermiogenesis in Mp*atg5-1^ge^* spermatids. Compared with wild-type spermatids, striking differences were detected at stages 1 and 2; the plastids in Mp*atg5-1^ge^* were complex in shape than the spherical wild-type plastids (**Figures 3A–E**). These results suggest that autophagy is involved in drastic morphological changes in the plastids during early spermiogenesis.

It has been reported that thylakoids are lost and starch deposition occurs as spermiogenesis proceeds, resulting in plastids mostly filled with starch granules in the spermatozoids of many plant species (Renzaglia and Garbary, 2001). Consistent with previous observations, the wild-type plastid at around stage 1-2 contained many starch granules that were observed as electron-dense structures using TEM. However, the plastids in Mp*atg5-1^ge^* spermatids contained significantly fewer starch granules than wild-type plastids, suggesting that autophagy may be involved in starch synthesis regulation during spermiogenesis (**Figures 3F, G**). These results suggest that autophagy also plays important roles in plastid reorganization, which is required for shaping plastid and starch synthesis during spermiogenesis, although its role in the regulation of the number of plastids is minor.

### The plastid is degraded by autophagy during spermiogenesis

**Figure 4:**
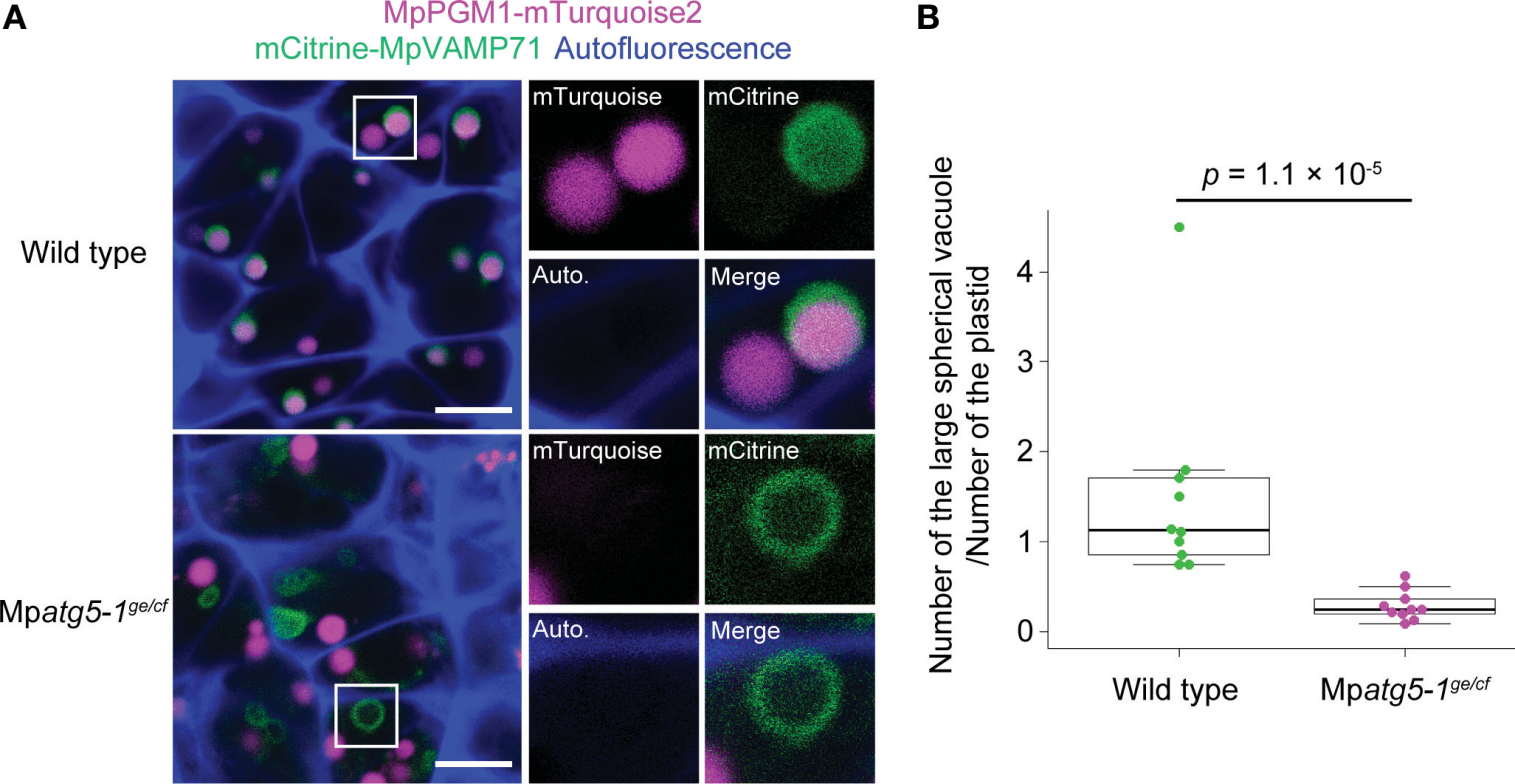
MpATG5-dependent vacuolar degradation of the plastid during spermiogenesis. **(A)** Confocal images of hand-sectioned antheridial cells of wild-type and Mp*atg5-1^ge/cf^* mutant plants co-expressing MpPGM1-mTurquoise2 (magenta) and mCitrine-MpVAMP71 (green). The autofluorescence probably from auronidin (Berland et al., 2019) is shown in blue. 10 images (22.49 μm × 22.49 μm) were obtained for each genotype, and representative images are presented. The right two panels show the enlarged images of the boxed regions. Scale bars = 5 μm. **(B)** The number of large spherical vacuoles observed in the confocal sections. The vacuole number was normalized by the plastid number, because most of wild-type and Mp*atg5* spermatozoids possess a single plastid (see **Figures 2**). Vacuoles and plastids in 10 images obtained in **(A)** were counted for each genotype. The boxes and solid lines in the boxes indicate the first and third quartiles and median, respectively. The upper and lower whiskers are drawn at the greatest value smaller than 1.5× the IQR above the third quartile and the smallest value greater than 1.5× the IQR below the first quartile, respectively. The *p* values obtained by the Wilcoxon rank-sum test is indicated.

We previously demonstrated that multiple organelles including mitochondria are degraded in the vacuole through autophagy during spermiogenesis in *M. polymorpha* (Norizuki et al., 2022). The observation that the autophagy-defective mutant exhibited impaired morphological transformation of the plastid and starch accumulation prompted us to investigate whether autophagy-dependent degradation of the plastid occurs during spermiogenesis. For this purpose, we investigated a plastid marker in spermatids, in which the vacuolar membrane was also visualized using mCitrine-MpVAMP71 (Kanazawa et al., 2016; Minamino et al., 2017). In wild-type spermatids, fluorescence from MpPGM1-mTurquoise2 was detected in the vacuole lumen, indicating that some of the plastid stroma was transported into the vacuole for degradation (**Figure 4A**). However, in Mp*atg5-1^ge/cf^* spermatids (Norizuki et al., 2022), the fluorescent signal from MpPGM1-mTurquoise2 was not detected in the vacuole (**Figure 4A**). We also noted that the number of the large spherical vacuole detected in confocal images was significantly decreased in Mp*atg5-1^ge/cf^* spermatids (**Figure 4B**). The mistargeting of MpPGM1-mTurquoise2 should not be due to the reduced number of the functional vacuole, because vacuolar targeting and degradation of the plasma membrane protein MpSYP12A and Golgi proteins occur in Mp*atg5-1^ge/cf^* spermatids similar to wild-type spermatids (Norizuki et al., 2022). These results indicate that a portion of the plastid is degraded by autophagy during spermiogenesis in *M. polymorpha*.

### Plastid DNA is eliminated from spermatids independent of autophagy

**Figure 5:**
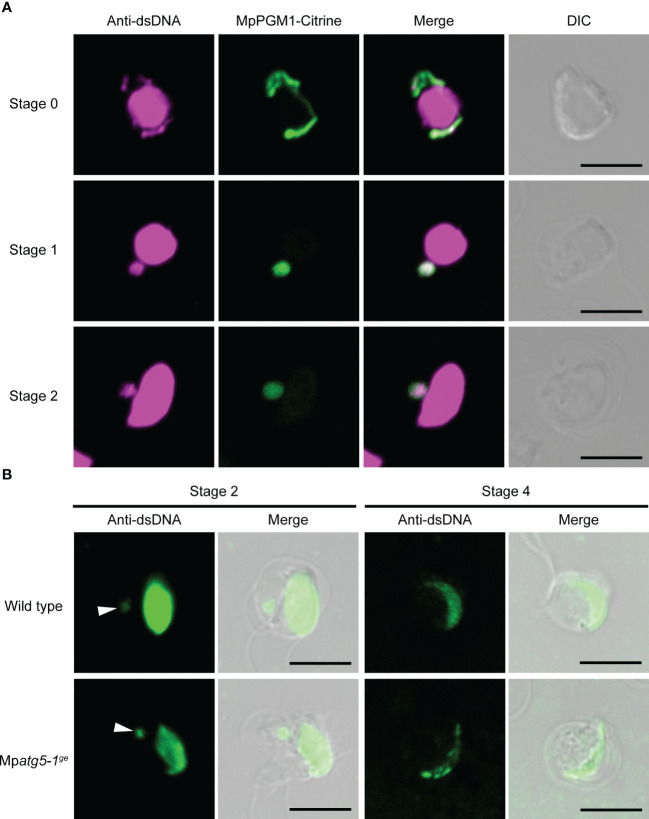
MpATG5-independent loss of plastid DNA during spermiogenesis. **(A)** Immunostaining of dsDNA (magenta) in wild-type antheridial cells expressing MpPGM1-Citrine (green). At least seven cells were observed at each stage and representative images are presented. Scale bars = 5 μm. DIC: differential interference contrast. **(B)** Immunostaining of dsDNA (green) in wild-type and Mp*atg5-1^ge^* spermatids. Arrowheads indicate signals from plastid DNA. As a secondary antibody, Alexa Fluor 546 **(A)** or Alexa Fluor Plus 488 **(B)** was used. At least three cells were observed and representative images are presented. Scale bars = 5 μm.

Unlike nuclear DNA, plastid and mitochondrial DNA are uniparentally inherited, with minor exceptions (Kuroiwa, 2010). In bryophytes, elimination of plastid DNA has been reported to occur during spermiogenesis in the liverwort *Dumortiera hirsuta* and the hornwort *Anthoceros punctatus* (Izumi and Ono, 1999; Shimamura et al., 1999). Based on our finding that a portion of the plastid is degraded *via* autophagy during spermiogenesis in *M. polymorpha* (**Figure 4**), we hypothesized that plastid DNA could be degraded through autophagy. To test this hypothesis, we detected organelle DNA using an dsDNA antibody. In addition to the strong signal in the nucleus representing nuclear DNA, we detected fluorescent signals in the cytoplasm of wild-type spermatids at stages 0–2. This signal overlapped with the plastid visualized using MpPGM1-Citrine, suggesting that this signal represents the plastid DNA (**Figure 5A**). Under our experimental conditions we did not detect mitochondrial DNA during spermiogenesis. In cells at stage 4, signal intensity from nuclear DNA was reduced compared with earlier stages, probably due to the restricted accessibility of the antibody to nuclear DNA resulting from chromatin compaction through the function of protamine (**Figure 5B**) (Kreitner, 1977; D’Ippolito et al., 2019). At this stage, the signal from plastid DNA was not detected, suggesting that elimination of plastid DNA occurs before stage 4 during spermiogenesis in *M. polymorpha* (**Figure 5B**). Contrary to our expectations, the removal of plastid DNA was also observed in Mp*atg5-1^ge^* spermatids (**Figure 5B**). This result indicates that autophagy is dispensable for plastid DNA removal during spermiogenesis in *M. polymorpha*.

## Discussion

In this study, we showed that autophagy is involved in the reorganization of plastids during spermiogenesis in *M. polymorpha*, especially during morphological transformation. Plastid degradation through autophagy has been intensively analyzed in *Arabidopsis thaliana*; it is divided into two types: a piecemeal type in which a portion of the plastid is degraded by autophagy (Ishida et al., 2008; Wang et al., 2013; Michaeli et al., 2014), and autophagic degradation of entire plastids (Wada et al., 2009; Izumi et al., 2017; Nakamura et al., 2018). These types of plastid degradation are both dependent on ATG5 activity (Ishida et al., 2008; Wang et al., 2013; Michaeli et al., 2014; Izumi et al., 2017; Nakamura et al., 2018) and responsible for normal stress responses (Izumi and Nakamura, 2018; Zhuang and Jiang, 2019; Wan and Ling, 2022); however, it remains unclear whether autophagy of plastids is involved in cellular differentiation. In this study, we showed that in *M. polymorpha*, ATG5-dependent autophagic degradation of a portion of the plastid occurs during differentiation from the spermatid to the spermatozoid.

During spermiogenesis in *M. polymorpha*, the loss of function of Mp*ATG5* did not substantially affect the number of plastids (**Figure 2A**), suggesting that piecemeal plastid degradation occurs in spermatids undergoing spermiogenesis. Although several cargoes for the piecemeal-type autophagic degradation of plastids have been reported (Izumi and Nakamura, 2018; Zhuang and Jiang, 2019; Wan and Ling, 2022), it remains unknown which components of the plastid are degraded by autophagy during spermiogenesis in *M. polymorpha*. Although plastid DNA is eliminated during spermiogenesis in *M. polymorpha*, autophagy is unnecessary for this process, indicating that plastid DNA is not a direct target of autophagy (**Figure 5**). Several factors that affect plastid morphology in somatic plant cells have been reported, including sugar content and osmolarity (Haswell and Meyerowitz, 2006; Stettler et al., 2009; Veley et al., 2012). As starch accumulation is reduced in the Mp*atg5-1^ge^* spermatid (**Figures 3F, G**), autophagy may regulate plastid morphology *via* degradation of the regulatory system for starch synthesis, which would affect sugar content and/or osmolarity in the plastid. However, a secondary effect due to the impaired reorganization of organelles other than the plastid cannot be ruled out, given the association of the plastid with other organelles, such as the nucleus and the posterior mitochondrion, during spermiogenesis (Carothers, 1975; Norizuki et al., 2022).

In addition to the plastid, our previous research has demonstrated that the endoplasmic reticulum, the Golgi apparatus, mitochondria, and peroxisomes are also degraded through autophagy during spermiogenesis in *M. polymorpha*. This autophagy-dependent reorganization is critical for spermatozoid function, such as motility and fertility. The degradation of mitochondria appears to occur before that of other organelles, indicating that the autophagic degradation of each organelle may be regulated differently (Norizuki et al., 2022). The degradation of the plastid must also be distinctly regulated from that of other organelles; unlike endomembrane organelles such as the endoplasmic reticulum and Golgi apparatus, which are fully removed from spermatids during spermiogenesis, the plastid is retained in the mature spermatozoid (**Figure 1A**; Minamino et al., 2022; Norizuki et al., 2022). Further studies will be required to fully understand the molecular mechanisms underlying the degradation of each organelle. Intriguingly, organelle elimination occurs after spermiogenesis in some bryophyte species; the plastid and the posterior mitochondrion have been observed to be removed from spermatozoids during movement in water after release from the antheridia in certain bryophytes, such as the liverwort *Blasia pusilla* and the moss Sphagnum (Manton, 1957; Sears, 1980; Renzaglia and Duckett, 1987). Thus, the elimination of the plastid and mitochondria occurs at multiple stages in bryophytes.

Autophagy is also required for spermiogenesis in organisms other than *M. polymorpha*, but its roles seem to be differentiated among eukaryotic lineages (Norizuki et al., 2020). Even in bryophytes, distinct effects of defective autophagy on plastid reorganization have been reported. Although a larger number of plastids is observed in autophagy-defective mutants of the moss *P. patens* (Sanchez-Vera et al., 2017), the plastid number was not markedly altered by the Mp*atg5-1^ge^* mutation in the liverwort *M. polymorpha* in our study (**Figure 3A**). The roles of autophagy in the reorganization of mitochondria and microtubule structures also differ between these species (Sanchez-Vera et al., 2017; Norizuki et al., 2022). These lines of evidence could reflect distinctly regulated organelle reorganization *via* autophagy among bryophytes, although bryophyte spermatozoids possess a shared organelle composition (Renzaglia and Garbary, 2001). Further analyses are needed to obtain insights into the shared and diversified mechanisms of organelle reorganization during spermiogenesis in bryophytes.

In conclusion, autophagy is important for morphological changes in plastids during spermiogenesis in *M. polymorpha*. Although plastids exhibit a pleiomorphic nature, it remains unclear how their complex morphology is controlled (Osteryoung and Pyke, 2014; Hanson and Conklin, 2020). Reorganization of the plastid during spermiogenesis in *M. polymorpha* should be a good model for analyzing the regulatory mechanisms of plastid morphology, with respect to not only the roles of autophagy but also to other cellular activities, given that the drastic morphological transformation occurs in a relatively short period in a synchronous manner in its antheridium.

## Data availability statement

The original contributions presented in the study are included in the article/**Supplementary Material**. Further inquiries can be directed to the corresponding author.

## Author contributions

TN performed the majority of the experiments. NM performed TEM experiments. TN and TU wrote the manuscript and MS and TU supervised the study. All authors contributed to the article and approved the submitted version.
