# Supplementary material for: Autophagy regulates plastid reorganization during spermatogenesis in the liverwort Marchantia polymorpha
Source: Front Plant Sci. 2023 Feb 9;14:1101983. doi: 10.3389/fpls.2023.1101983 (PMC9947651; doi:10.3389/fpls.2023.1101983)
Supplement: Supplementary file 1 [file DataSheet_1.pdf]

Supplementary Material

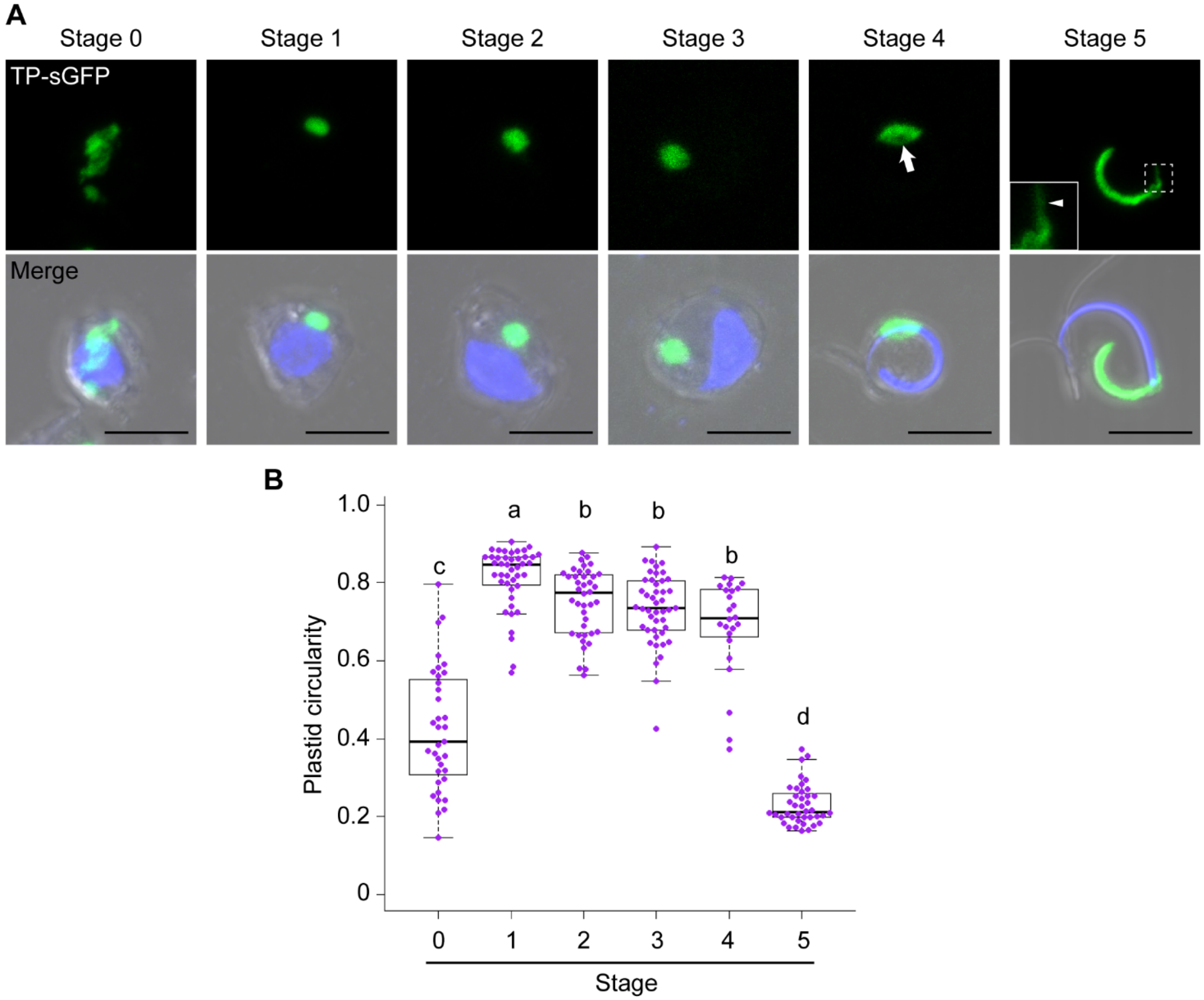

Supplementary Figure 1. Plastid reorganization during spermiogenesis in *M. polymorpha*. (**A**) Maximum-intensity projection images of cell wall-digested antheridial cells and a spermatozoid at each developmental stage expressing TP-sGFP (green). A total of 35 stage-0, 43 stage-1, 40 stage-2, 45 stage-3, 24 stage-4, and 40 stage-5 cells were observed; representative images are presented. The nuclei were stained with Hoechst 33342 (blue). The arrow and arrowhead indicate the region of the weak signal from TP-sGFP and tubular extension from the plastid body, respectively. Scale bars = 5 μm. (**B**) The circularity of the plastid calculated in the same sets of samples analyzed in (A). The boxes and solid lines in the boxes indicate the first quartile and third quartile and the median, respectively. The upper and lower whiskers are drawn at the greatest value smaller than 1.5× the interquartile range (IQR) above the third quartile and the smallest value greater than 1.5× the IQR

below the first quartile, respectively. Different letters denote significant differences based on the Steel–Dwass test ($p < 0.05$).

Supplementary Table 1. The list for transgenic plants used in this study

Transgenic plants	Reference
$_{pro}$Mp*EF1α:*Mp*PGM1-Citrine*	This study
$_{pro}$Mp*EF1α:TP-sGFP*	This study
Mp*atg5-1^{ge}*	Norizuki et al., 2019
Mp*atg5-1^{ge}*/$_{pro}$Mp*EF1α:*Mp*PGM1-Citrine*	This study
$_{pro}$Mp*VAMP71:mCitrine*-Mp*VAMP71*	Minamino et al., 2017
$_{pro}$Mp*VAMP71:mCitrine*-Mp*VAMP71*/$_{pro}$Mp*EF1α:*Mp*PGM1-mTurquoise2*	This study
Mp*atg5-1$^{ge/cf}$*/$_{pro}$Mp*VAMP71:mCitrine*-Mp*VAMP71*	Norizuki et al., 2022
Mp*atg5-1$^{ge/cf}$*/$_{pro}$Mp*VAMP71:mCitrine*-Mp*VAMP71*/$_{pro}$Mp*EF1α:*Mp*PGM1-mTurquoise2*	This study

Supplementary Table 2. The primer list

	Primer 1 (5´->3')	Primer 2 (5´->3')
pENTR Mp*PGM1*	**CACCATGGCGTTCTCCGCAGCA**	**CGTAATTACTGTTGGCTTGGTTCG**
mTurquoise2 (*Asc*I)	**AAGGGTGGGCGCGGGGTGTCTAAGGGTGAGGAACTCTTC**	**AGCTGGGTCGGCGCGCTATTTGTAAAGCTCATCCATTCCGAG**
*TP*_Mp*SIG2*	CACCATGGCGGTTGTTGCTACGAG	CGCCCTTGCTCACCATGGTGCCGTCGACCCCGGCAGATGCAGCAGTGAC
sGFP	CACCATGGTGAGCAAGGGCGAGGAG	TTACTTGTACAGCTCGTCCATGC
TP-sGFP	CACCATGGCGGTTGTTGCTACGAG	TTACTTGTACAGCTCGTCCATGC
